# Effects of awake caudal anesthesia on mean arterial blood pressure in very low birthweight infants

**DOI:** 10.1186/s12871-020-01094-8

**Published:** 2020-07-20

**Authors:** Frank Fideler, Michael Walker, Christian Grasshoff

**Affiliations:** 1 Departmnt of Anesthesiology and Intensive Care Medicine, University Hospital Tuebingen, Tübingen, Germany; 2 Clinic for Anesthesiology, Intensive, Emergency- and Pain-Therapy, Ludwigsburg, Germany

## Abstract

**Background:**

Intraoperative blood pressure is a relevant variable for postoperative outcome in infants undergoing surgical procedures. It is therefore important to know whether the type of anesthesia has an impact on intraoperative blood pressure management in very low birth weight infants. Here, we retrospectively analyzed intraoperative blood pressure in very low birthweight infants receiving either awake caudal anesthesia without sedation, or caudal block in combination with general anesthesia, both for open inguinal hernia repair.

**Methods:**

Ethical approval was provided by the University of Tuebingen Ethical Committee on 05/29/2018 with the project number 403/2018BO2. Patient records of infants admitted by the neonatologist (median age at birth 31.1 ± 3.5 weeks, median weight at birth 1240 ± 521 g) which were scheduled for inguinal hernia repair were retrospectively evaluated for the course of mean arterial blood pressure and perioperative interventions to stabilize blood pressure. A total of 42 patients were included, 16 patients (11 boys, 5 girls) received awake caudal anesthesia, 26 patients (22 boys, 4 girls) a combination of general anesthesia and caudal block.

**Results:**

Approximately 3% of the measured mean arterial blood pressure values in the caudal anesthesia group were below a critical margin of 35 mmHg, in contrast to 47% in the combined anesthesia group (*p* < 0.001). Patients in the latter group showed a significantly larger drop of mean arterial blood pressure below 35 mmHg (4.7 ± 2.7 mmHg vs. 1.9 ± 1.6 mmHg; *p* < 0.005) and a significantly longer time of mean arterial blood pressure below 35 mmHg (25.6 ± 26.0 min vs. 0.9 ± 2.3 min; *p* < 0.001), although they received more volume and vasopressor boluses for stabilization (27 ± 14.8 ml vs. 10 ± 4.1 ml; *p* < 0.01 and 0.15 ± 0.06 ml vs. 0 ml of cafedrine/theoadrenaline; *p* < 0.001).

**Conclusions:**

The study indicates that the use of caudal block as stand alone procedure for inguinal hernia repair in very low birthweight infants might be advantageous in preventing critical blood pressure drops compared to a combination of caudal block with general anesthesia.

## Background

The outcome relevance of adequate blood pressure in pediatric surgery has been lately emphasized by several authors [1, 2]. For example, McCann et al. have reported a case series with pediatric patients suffering from infantile postoperative encephalopathy after the occurrence of intraoperative hypotension [3]. This raises the question whether the type of anesthesia has an impact on the need for active intraoperative blood pressure management. To keep blood pressure stable Marhofer et al. promoted the use of caudal and epidural anesthesia under sedation as one possibility to minimize the use of general anesthetic drugs [4]. The authors argued that by the use of regional techniques an avoidance of opioids during the perioperative period might help to reduce cardiorespiratory depression and improve gut function. However, to date there is little evidence supporting the concept that cardiocirculatory parameters in neonates are less impaired during caudal anesthesia compared with general anesthesia. So far, Ing et al. have demonstrated that spinal anesthesia performed in healthy infants undergoing pyloromyotomy results in reduced intraoperative blood pressure changes from baseline compared to general anesthesia [5]. Regarding caudal anesthesia, we already know from previous studies that arterial blood pressure is not altered by caudal anesthesia in children receiving general anesthesia [6], a finding that was supported by a prospective study in neonates by Deng et al. [7].

Thus, to determine whether the use of caudal block as a stand alone method may be advantageous in preventing critical blood pressure drops in the care of very low birthweight infants, we performed a retrospective analysis comparing patients with either awake caudal anesthesia without sedation (CA), or with a caudal block in combination with general anesthesia (GA + CA).

## Methods

Ethical approval for this study was provided by the University of Tuebingen Ethical Committee on 05/29/2018 with the project number 403/2018BO2 (Chairperson Prof D. Luft). Anesthesia in both groups was performed by two senior anesthetists with more than 5 years of experience in the care of very low birthweight infants. Both anesthesiologists provided anesthetics of both types. The decision regarding the technique used was made up to their clinical preference.

### Patients’ characteristics

Patient records of infants admitted by the neonatologist which were scheduled for inguinal hernia repair between 01/01/2012 and 05/22/2014 were retrospectively evaluated. All children with hernia repair during that period have been evaluated (*n* = 52). We included only children who underwent an isolated inguinal hernia repair and who had no concomitant diseases (*n* = 42). Patients with congenital organ disorders like cardiovascular disease that could affect mean arterial pressure were not included into the study.

### Definition of target size

In order to define a critical margin of mean arterial blood pressure we refer to a study by Rhondali et al. who conducted a retrospective analysis to compare the data of cerebral blood flow (CBF) and brain oxygenation in 180 children younger than 6 months. The authors stated that the mean arterial pressure is a good proxy of cerebral perfusion as they found that the CBF decreased proportionally with cerebral perfusion pressure. They regarded a mean arterial pressure beyond 35 mmHg during anesthesia to be safe and sufficient [8]. Therefore, we defined the incidence of a mean arterial pressure below 35 mmHg as the primary target. Secondary target sizes were the extent of fall of mean arterial pressure below the limit of 35 mmHg as well as the cumulative time per patient below that limit.

### Data handling

Vital parameters were automatically recorded by use of a patient data management system (IntelliSpace Critical Care and Anesthesia (ICCA); Version H.02.01.001, Philips®). Evaluation of the data was carried out manually. Statistical analysis was performed by GraphPadPrism® 5.02. The Shapiro-Wilk’s test for normality was used to check for normal distribution. Normal distributed groups were compared with the 2-sample 2-sided t-test. Otherwise the Mann-Whitney-U Test was used.

## Results

In this study a retrospective analysis was performed in very low birthweight neonates (birthweight 1000 – 1500 g) receiving inguinal hernia repair. Two groups were compared: neonates with awake caudal anesthesia without sedation (CA) and neonates with a caudal block in combination with general anesthesia (GA + CA). At our institution we left treating preterm neonates for inguinal hernia repair without caudal block over a decade, since we rated the well-known advantages of regional anesthesia as so convincing [9, 10]. Therefore, a third study arm (general anesthesia alone) was not created. Table 1 shows that baseline characteristics are well balanced between both groups of very low birthweight infants. Patients who received CA had a lower body weight at the time of the surgical procedure. However, the difference is tiny and we therefore did not adjust for any baseline characteristic in our analysis. Median post menstrual age at birth was 31.6 weeks (GA + CA) vs 30.1 (CA). Post menstrual age at the time of operation was 39.8 weeks (GA + CA) vs 37.8 (CA). Sixteen patients (11 boys, 5 girls) received a CA, while 26 patients (22 boys, 4 girls) received a GA + CA.

**Table 1:** Baseline characteristics of very low birthweight infants receiving open inguinal hernia repair. Sixteen patients (11 boys, 5 girls) received a caudal anesthesia as a stand-alone method, 26 patients (22 boys, 4 girls) a combination of a general anesthesia with a caudal anesthesia. The baseline characteristics are well balanced between both groups (Mann-Whitney-U test)

	GA + CA (*n* = 26)			CA (*n* = 16)			
	Median	25–75% percentile	Min - Max	Median	25–75% percentile	Min - Max	*P*
birthweight [g]	1390	778–1850	560–2450	1220	869–1593	570–2150	0.32
PMA at birth [weeks]	31.57	29.68–34.07	25.57–38.86	30.07	27.00–32.72	24.29–34.29	0.11
Weight at OR [g]	2990	2375–3400	2000–3600	2400	2149–3050	2000–3600	0.05
PMA at OR [weeks]	39.79	36.86–41.61	35.14–48.29	37.79	36.40–39.71	35.43–43.71	0.11
Duration of surgery [min]	25.5	19.5–49.0	14.0–66.0	29.5	19.75–40.0	8.0–66.0	0.9

*PMA* Post menstrual age, *OR* Operation

**Fig. 1:**
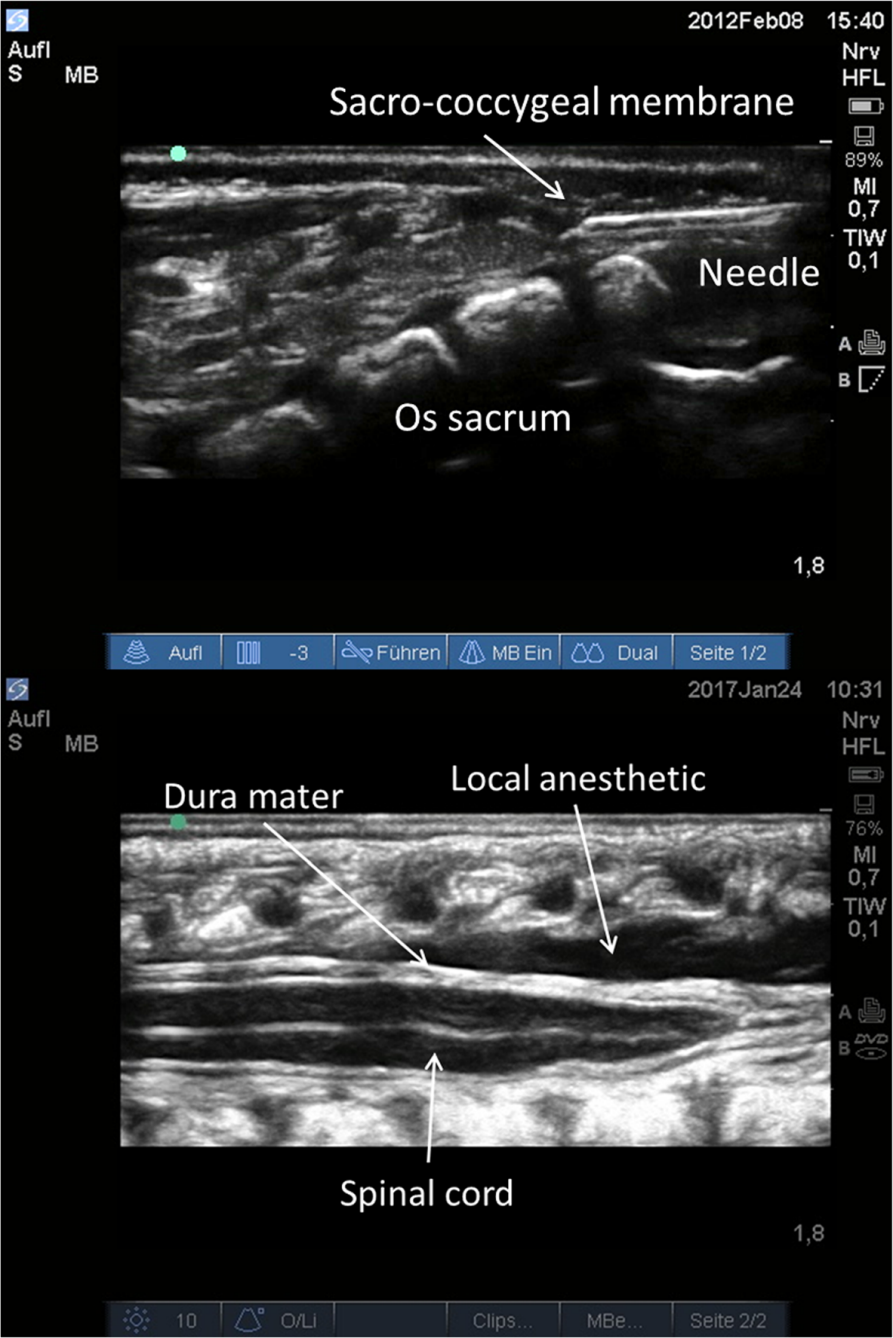
Ultrasound images of the implementation of caudal anaesthesia in very low birth weight infants scheduled for open inguinal hernia repair. Caudal anaesthesia was implemented in infants placed in a lateral decubitus position with the left side down. Correct needle placement and distribution of the local anaesthetic was observed in all patients as visible on the ultrasound image and led to a surgically sufficient analgesia. Both pictures show a long axis view of the spinal canal. **a** Puncture with an Epican®Paed25G was performed in an in-plane technique by means of a SonoSite® M-Turbo with linear probe (5–13 MHz). **b** The ultrasound picture in in-plane technique demonstrates the spread of the local anaesthetic (ropivacaine 0.375%; 1 ml/kg)

Caudal anesthesia was performed in patients in a lateral decubitus position with left side down. The puncture site was preoperatively anesthetized with EMLA®. After implementation of an intravenous infusion and monitoring of vital parameters the patient was placed in a lateral decubitus position with left side down. An Epican® Paed25G was guided by a SonoSite® M-Turbo with linear probe (5–13 MHz) in long axis in-plane-technique and the needle tip advanced under direct visualization. After negative aspiration, local anesthetic (ropivacaine 0.375%; 1 ml/kg) was applied and its spread assessed by ultra sound (Fig. 1).

The patients received no further medication until the end of anesthesia.

**Fig. 2:**
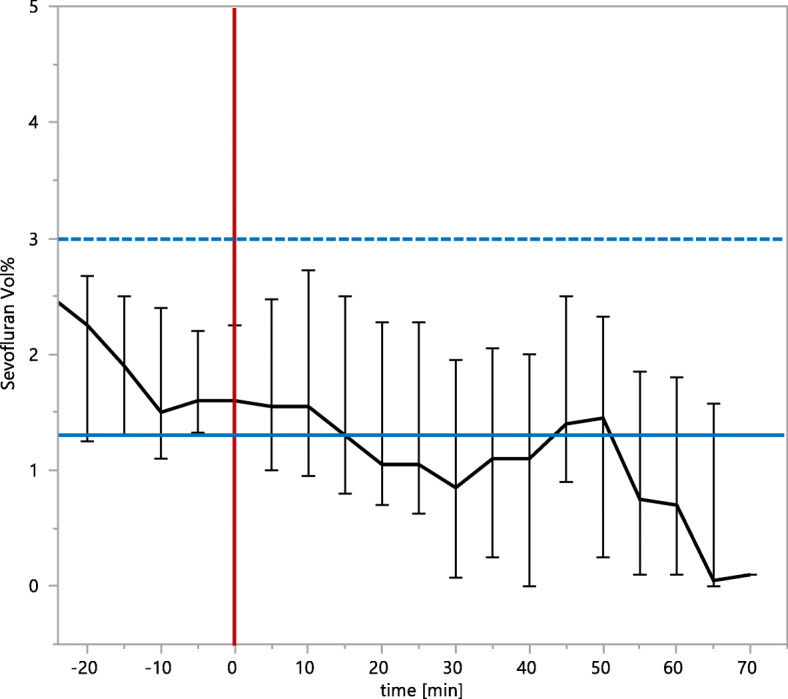
Time course of the median sevoflurane concentration (median ± interquartile range) calculated from patients in the group of combined general- and caudal anesthesia. The time point “0 min” indicates the start of the surgical procedure and is marked by the vertical red line. The dashed blue line indicates a sevoflurane concentration of 3.0 vol% corresponding to 1 MAC. The blue line represents the median sevoflurane concentration of 1.3 vol% during the maintenance phase of general anesthesia

General anesthesia was either induced by mask ventilation with sevoflurane or by intravenous injection of 2–4 mg/kg propofol. In 12 patients the trachea was intubated, and in 14 patients the airway was secured by use of a laryngeal mask (Ambu® AuraOnce™). In order to facilitate intubation of the trachea the muscle relaxant vecuronium (0.1 mg/kg) was applied. General anesthesia was maintained with sevoflurane (0.5–2.9 Vol% corresponding to 0.3–1 MAC), 13 patients received additionally remifentanil (0.1–0.3 μg/kg/min) (Fig. 2, Table 2) After induction of general anesthesia, a caudal block was applied as described above. In contrast to patients with CA, the local anesthetic was used in a concentration of 0.2% (1 ml/kg). This anesthetic practice followed in-house standard operating procedures and was not changed within the study period. As a result, all caudal blocks were successful and there were no complications or side effects registered.

**Table 2:** The table summarizes the doses and ranges of anesthetic agents used in the group of combined general- and caudal anesthesia

Anesthetic agent	i.v. Induction (n)	Mask Induction (n)	maintenance of anesthesia (n)
Propofol [mg/kg]	2–4 (11)	–	–
Sevofluran [vol%]	–	0.6–7.3 (15)	0.5–2.9 [mean 1.3] (26)
Remifentanil [μg/kg/min]	–	–	0.1–0.3 (13)
Vecuronium [mg/kg]	0.1 (4)	–	–

Patients in both groups received an intravenous isotonic full electrolyte solution including 1 % glucose with an infusion rate of 10 ml/kg/h. Basic vital parameters like blood pressure, heart rate, oxygen saturation and body temperature were continuously monitored during the operation and automatically recorded.

In accordance with the American Heart Association (AHA) recommendations for blood pressure measurement in children, cuff bladder width was at least 40% of the arm circumference halfway between the olecranon and acromion. The cuff covered at least 80% of the arm circumference.

**Fig. 3:**
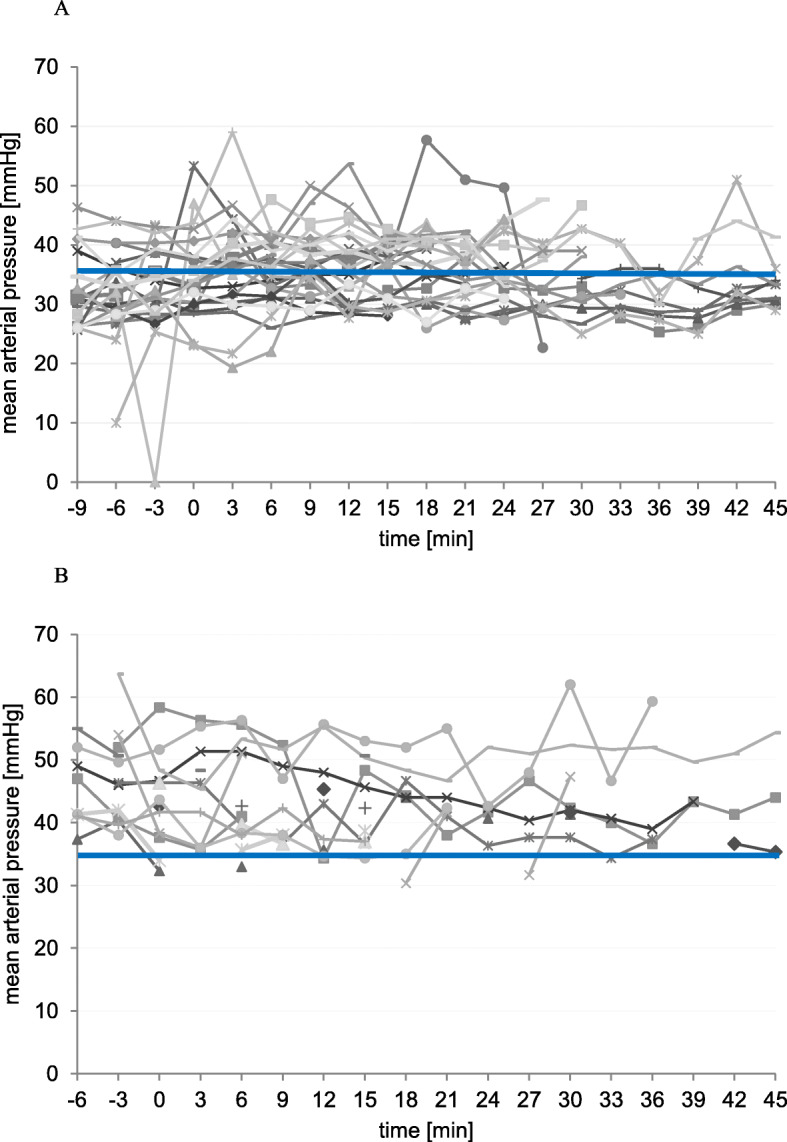
The graph depicts the individual courses of mean arterial blood pressure in very low birth weight infants during open inguinal hernia repair. Each character represents a single patient. The surgical procedure started at the time point 0 min. For easier interpretation of the data a blue line was drawn at 35 mmHg, since this value marked the threshold that was defined to be safe according to Rhondali et al. [8] **a** The graph visualizes that in the group of combined general- and caudal anesthesia approximately half of the mean blood pressure values measured during inguinal hernia repair were below the critical threshold of 35 mmHg. **b** In contrast to the patients in the group of combined general- and caudal anesthesia only 3% of all mean blood pressure values measured in the caudal anesthesia group underran the critical margin of 35 mmHg

In Fig. 3 we plotted the individual courses of the patients’ mean arterial blood pressure. According to Rhondali et al. we defined a mean arterial pressure above 35 mmHg to be safe in this population [8]. The mean recording period was 47.4 ± 24.3 min. As demonstrated in Fig. 3a, approximately half of the mean blood pressure values were below the defined critical threshold of 35 mmHg in the GA + CA group, whereas this was the case in only 3% in the CA group (Fig. 3b). 57.7% of the patients in the GA + CA group underran the critical margin of 35 mmHg compared to 18.8% in the CA group.

**Fig. 4:**
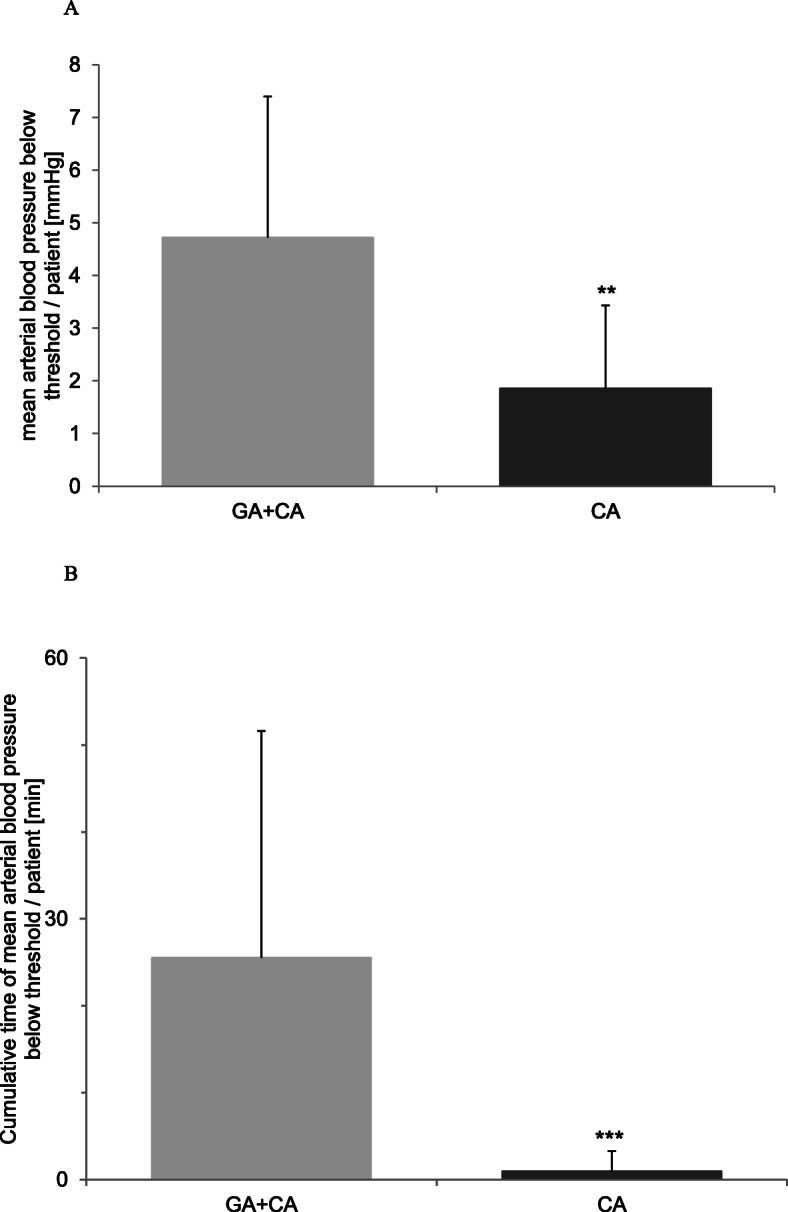
Evaluation of potentially critical episodes (mean arterial blood pressure values below 35 mmHg) in both groups of very low birth weight infants receiving open inguinal hernia repair. **a** The figure demonstrates that the extent to which the critical limit of 35 mmHg was underran is significantly greater in the group of combined general- and caudal anesthesia (GA + CA) (**; *P* < 0.01). **b** Analysis of the mean cumulative time per patient with a mean arterial pressure value below 35 mmHg. Patients in the group of combined general- and caudal anesthesia (GA + CA) spent significantly longer time with mean arterial pressure below 35 mmHg compared to patients in the caudal anesthesia (CA) group (***; *P* < 0.001)

In the following step we quantified these episodes with a critical mean arterial blood pressure. As depicted in Fig. 4, both severity of MAP (Fig. 4a) (4.7 ± 2.7 mmHg vs 1.9 ± 1.6 mmHg) and mean cumulative time per patient of MAP below 35 mmHg (Fig. 4b) (25.6 ± 26.0 min vs 0.9 ± 2.3 min) were significantly higher in the GA + CA group.

Intraoperative interventions to stabilize blood pressure are summarized in Table 3. The application of full electrolyte solution (Jonosteril®) or a vasopressor (Akrinor®; cafedrine/theoadrenaline) was at the discretion of the anesthesiologist. In the GA + CA group 15 out of 26 patients (57.7%) received a volume bolus in contrast to 3 out of 16 patients (18.8%) in the CA group. The median cumulative amount of bolus volume applied to patients in this group was significantly lower (10 ± 4.1 ml vs. 27 ± 14.8 ml; p = 0.0015). In accordance with this, vasopressor application was less frequent in the caudal anesthesia group (0% versus 19%).

**Table 3:** depicts intraoperative interventions to raise blood pressure in very low birthweight children receiving open inguinal hernia repair. Full electrolyte solution boli (Jonosteril®) or vasopressor boli (Akrinor®; cafedrine/theoadrenaline) were administered at the discretion of the anaesthesiologist (Mann-Whitney-U test)

		GA + CA (n = 26)	CA (*n* = 16)	*P*
Fluid bolus	Patients n [%]	15 [57.7]	3 [18.8]	0.0046
	Average fluid bolus [ml/patient] [mean]	27 ± 14.8	10 ± 4.1	0.0015
Cafedrine / theoadrenaline	Patients n [%]	5 [19.2]	0 [0]	< 0.001
	Average vasopressor bolus [ml/patient] [mean]	0.15 ± 0.06	0	< 0.001

## Discussion

In this retrospective study we evaluated the course of intraoperative blood pressure in very low birthweight infants who received either caudal anesthesia, as a stand alone method, or a combination of general anesthesia, and caudal anesthesia, both for inguinal hernia repair. Anesthesia in both groups was performed by senior anesthetists with more than 5 years experience in the care of very low birthweight infants. As reported in the results section we did not investigate the effects of general anesthesia as a stand alone method, although general anesthesia may still be used in many institutions for this type of surgery. In our opinion, the two study arms are justifiable since the aim of our study was to find out potential advantages of caudal anesthesia as a stand-alone method in preterm neonates. Furthermore, we know from literature that arterial blood pressure is not altered by caudal anesthesia in children receiving general anesthesia (Larousse et al. 2002), a finding that was supported by a prospective study in neonates by Deng et al. in 2008 [6, 7].

The most striking result is that the mean blood pressure was significantly lower in the GA + CA group although there were more pharmacological interventions like application of vasopressors or volume boluses. It is of even more importance that approximately 47% of the measured mean arterial blood pressure values in that group were below a critical margin of 35 mmHg, in contrast to only 3% in the CA group and that the cumulative time below 35 mmHg per patient was significantly longer. A safe lower limit of blood pressure in infants is still a matter of debate. The incidence of hypotension in preterm neonates seems to be inversely related to gestational age and birth weight [11]. General anesthesia can contribute to hypotension, and thereby cause low cerebral perfusion [1, 8]. This seems to be less critical in preterm neonates who do not undergo surgical intervention since Alderliesten et al. described that a mean arterial blood pressure less than gestational age in weeks was not associated with lower neurodevelopmental outcome scores [11]. On the other hand McCann et al. reported on 6 infants who underwent elective surgery and developed postoperative encephalopathy, which had features most consistent with intraoperative cerebral hypoperfusion [3]. However, in a retrospective analysis in children younger than 6 months conducted by Rhondali et al., the authors compared data of two studies investigating the impact of sevoflurane anesthesia on cerebral blood flow by transcranial Doppler [12] and on brain oxygenation by NIRS [13]. The authors reported that in healthy infants scheduled for short procedures, the mean arterial pressure is a good surrogate parameter of cerebral perfusion. Even more Rhondali et al. stated that maintaining the mean arterial pressure above 35 mmHg during anesthesia is safe and sufficient [8], and we adopted this value as a lower threshold. It is important to mention that general anesthetics were administered at low concentrations, e.g. sevoflurane was dosed by approximately 0.5 MAC (median 1.3 vol%) during the maintenance phase of general anesthesia which is far less compared to sevoflurane concentrations reported in other studies (Fig. 2) [13, 14]. Even for sedation the application of either 8 vol% sevoflurane or up to 4 mg/kg propofol during the initiation of a caudal block were reported [15, 16]. Thus it can be estimated that with a less cautious use of general anesthetics even more episodes of low mean arterial pressure can be expected.

In very low birthweight infants we use caudal anesthesia rather than spinal anesthesia since the success rates are significantly higher [14, 15, 17, 18]. The quality of caudal blockade provided adequate surgical conditions in all cases although a lack of sufficient motor blockage was often considered to be disadvantageous [17, 19]. For this ropivacain 0,375% was used in the CA group without any side effects. In contrast to spinal anesthesia, the implementation of a caudal block is technically less difficult and became even more straightforward by use of ultrasound control since puncture and correct spread of the local anesthetic can be tracked visually, especially when the needle is visualized in long axis in a sagittal plane [20, 21]. Another advantage is the chance to identify anatomical anomalies like for example a terminal myelocystocele, a myelomeningocele or a tight filum terminale syndrome [22] before puncture.

The retrospective analysis suffers from some limitations like the limited number of patients being anesthetized by only two anesthetists or that an association of intraoperative hypotension to general anesthetics like remifentanil or sevoflurane cannot be derived. Likewise no sequelae could be recorded in this retrospective study. However, the anesthetists each had at least 5 years of experience in pediatric anesthesia and were aware of keeping the mean arterial pressure above 35 mmHg which was difficult in the group of combined general- and caudal anesthesia in spite of the use of volume boluses and vasopressors and resulted in significantly more episodes of critically low mean arterial blood pressure. 

## Conclusion

In summary this preliminary study provides support for the use of caudal block as a stand alone method for the care of very low birthweight infants as it shows advantages in preventing critical blood pressure drops compared to a combination of caudal block with general anesthesia.

## Data Availability

The datasets used and/or analysed during the current study are available from the corresponding author on reasonable request.

## Abbreviations

AHA: American heart association
CA: Awake caudal anesthesia without sedation
CBF: Cerebral blood flow
g: Gram
G: Gauge
G1%: Glucose 1%
GA + CA: General anesthesia with caudal block
ICCA: IntelliSpace Critical Care and Anesthesia information system
MAC: Minimum alveolar concentration
MAP: Mean arterial blood pressure
MHz: Megaherz
ml: Milliliter
mmHg: Millimeter mercury column
NIRS: Near-infrared spectroscopy
OR: Operation
PMA: Post menstrual age
vs: Versus
